# Individual and organisational factors in the psychosocial work environment are associated with home care staffs’ job strain: a Swedish cross-sectional study

**DOI:** 10.1186/s12913-022-08699-4

**Published:** 2022-11-26

**Authors:** Susanne Assander, Aileen Bergström, Helen Olt, Susanne Guidetti, Anne-Marie Boström

**Affiliations:** 1 Department of Neurobiology, Caring Sciences and Society, Division of Occupational Therapy, Karolinska Institutet, Stockholm, Sweden; 2 Department of Neurobiology, Care Sciences and Society, Division of Nursing, Karolinska Institutet, Stockholm, Sweden; 3 Theme Women´S Health and Allied Health Professionals, Medical Unit Occupational Therapy and Physiotherapy, Karolinska University Hospital, Stockholm, Sweden; 4 Theme Inflammation and Aging, Karolinska University Hospital, Stockholm, Sweden; 5 Research & Development Unit, Stockholms Sjukhem, Stockholm, Sweden

**Keywords:** Allied Health Personnel, Aging, Community Health Services, Delivery of Health Care, Health Service Research, Nursing, Organisations, Personnel Management, Reablement

## Abstract

**Background:**

Home care staff (HCS) provide essential service to enable older adults to age in place. However, unreasonable demands in the work environment to deliver a safe, effective service with high quality has a negative impact on the individual employee’s well-being and the care provided to the older adults. The psychosocial work environment is associated with employees´ well-being, although, knowledge regarding which individual and organisational factors that contribute to job strain for HCS is limited. These factors need to be identified to develop targeted interventions and create sustainable work situations for HCS. This study aimed to explore how HCS´s perceived job strain is associated with, and to what extent can be explained by, individual and organisational factors of the psychosocial work environment and psychosomatic health.

**Method:**

An explorative cross-sectional questionnaire survey design was used in a large Swedish county. Five home care agencies with a total of 481 HCS were asked to respond to a questionnaire regarding their perceived level of job strain (Strain in Dementia Care Scale), psychosocial work environment (QPS_Nordic_^34+^), and psychosomatic health (Satisfaction with Work Questionnaire). Multiple linear regression (MLR) analyses were conducted to explore the association between job strain and individual and organisational factors.

**Results:**

In total, 226 (46%) HCS responded to the questionnaire. Both individual and organisational factors were significant predictors of job strain and explained a variance ranging between 39 to 51% (*p* = 0.001). The organisational factor job demand and the individual factor feeling worried and restless was most frequently represented in these MRL models*.* A higher job strain was also associated with adverse outcomes regarding leadership, organisational culture and climate, and control at work.

**Conclusion:**

This study indicates that there is an intertwined complexity of individual and organisational factors that are associated with the HCS´s perception of job strain. Implementation of new multidimensional work strategies, such as a reablement approach, could support the development of efficient strategies for HCS and reduce the level of job strain. Policy changes for the provision of home care are also needed to support the development of a sustainable and healthy psychosocial work environment.

**Supplementary Information:**

The online version contains supplementary material available at 10.1186/s12913-022-08699-4.

## Background

To enable ageing in place for older adults, the provision of home care can be crucial. However, with an increasing ageing population in combination with an estimated shortage of nurse assistants and nurse aides in the home care workforce [1, 2], the health and social care welfare system will require financial resources as well as an increase of employees [1, 3, 4]. To retain and attract new employees within home care, in particular nurse assistants and nurse aides, it is of essence to develop a sustainable work environment that also is attractive. However, before a new sustainable work environment can be developed, information and understanding about the home care staffs’ (HCS) current work situation is required. Therefore, we want to explore how HCS perceives their psychosocial work environment and factors in the work environment that are associated with job strain.

In Sweden, home care for older adults is subsidised and provided by both public and for-profit organisations. The purpose with home care is to provide support in activities of daily living (ADL), social aspects, and medical distribution [1]. Nurse assistants and nurse aides are commonly employed within home care in Sweden, and women in this cohort are overrepresented (< 80%). The HCS´s work is independent, where the staff has limited collaboration with colleagues and managers as they perform their work in the homes of their clients´ [1, 5]. This creates demands on the individual worker to manage time, assess necessary skills and resources to support each client, and having to rely on one’s own competence and capacity [6]. Even so, to be employed as HCS in Sweden today, limited or no formal education within health and care is required, and formal education is usually equal to secondary school level [7, 8].

Throughout the last decades, organisational and regulatory changes have occurred within the home care service system in Sweden [1, 9]. Some of these changes have had negative consequences and increased the strain on HCS to uphold a sustainable service, which impacts both the individual and organisational psychosocial work environment [9]. One example is the expanded amount of tasks assigned to the HCS in combination with organisational restrictions, which have led to an increased workload that requires greater competencies to provide expected and proficient care [1, 5, 10, 11].

The psychosocial work environment and the organisational structure are multifaceted phenomena that are significantly associated with the health and well-being of care staff in different settings [7, 12–15]. There are well-established relationships regarding demand, resource, and support [16, 17] and how this impacts the work situation and health for the staff [18–20]. The composition and balance of the psychological work environment and the organisational structures, empowerment, and good management, are essential parts to prevent physical and psychological ill-health and can increase the job satisfaction for HCS [1, 5, 21–24]. In contrast to job satisfaction, job strain is a concept that deals with the perception of stress and strain related to work and reflects the perceived burden of the work environment [25]. Research targeting a diversity of occupations has identified that job strain is related to several factors in the work environment, such as leadership, conflicts at work, education and work experience [25–28]. In addition, experiencing high job demands, working independently without appropriate support, feeling out of control, or being unable to manage issues during work can further influence the perceived levels of job strain [16, 26, 27]. Combined, these issues increase the risk to negatively impact a person’s well-being and lead to health problems such as insomnia, increased incidences of stress and burnout, psychological disruptions, or long-term sick-leave [7, 10, 14, 23, 26, 27, 29].

Research targeting job strain for nurse assistants and nurse aides has mainly focused on those working in the context of residential nursing homes rather than those working within home care [6, 13, 25, 28]. In residential nursing homes, personal factors regarding level of education and feeling worried and stressed, in combination with work-related factors of working day time, and insufficient organisational and environmental support, caring climate, and leadership, have been associated with high job strain [14, 29]. The consequences of high job strain does not only negatively affect the individual but it also impacts the organisation in terms of high turnover rates [1, 29]. Subsequently, the older adult who is receiving the service can also be affected in terms of unattainable relationships with the staff and reduced quality of the received care, which could lead to a decreased quality of life [22, 29, 30].

Within a changing welfare organisation that demands stricter requirements for the provision of home care, and with tasks that command a higher level of competencies for the HCS, it is essential to investigate which individual and organisational factors of the psychosocial work environment that may contribute to a higher or lower job strain. There is limited research regarding job strain within the setting of home care as well as which factors that are associated with job strain for HCS. Hence, by gaining knowledge regarding which individual and organisational factors that impact the HCSs’ perceived job strain, more efficient and appropriate interventions could be developed to create a sustainable work situation and thereby facilitate the possibility to have a healthy and satisfied workforce.

The aim of this exploratory study is twofold; 1) to explore how HCS perceive their level of job strain, and 2) to examine how job strain is associated with, and to what extent job strain can be explained by, individual and organisational factors of the psychosocial work environment and psychosomatic health factors.

## Methods

This is an explorative study using a cross-sectional questionnaire survey design. Descriptive statistics were used to describe characteristics of the participating HCS, as well as levels of HCSs’ perceived job strain, their psychosocial work environment, and psychosomatic health factors. Inferential statistics were used for pre-analysis and to explore individual and organisational factors that were associated with job strain by conducting multiple linear regression (MLR) analysis.

### Setting and sample

Via the research & development units in Stockholm county, 26 municipalities were invited to participate in the study. Five of the invited municipalities agreed to participate, and within these municipalities, five home care agencies (public and for-profit) consisting of 17 home care units agreed to partake. Agencies that were included met the following criteria 1) having at least 30 employees (home care staff) who had been employed at least three months and had a contract equalling ≥ 50% employment (20 h/week), and 2) provide home care service to at least 30 older adults.

In total, 481 HCS employed at these units were requested to respond to the questionnaire.

### Instruments

The last author (A-M.B) compiled a survey that consisted of three standardised instruments (described below) to assess the dependent variable job strain and the independent individual and organisational variables.

The instruments were chosen as they focus on job strain, work environment, and perceived health. They are all considered to be concise and easy to administrate and have previously been used with diverse professions, including HCS, in a Swedish context. None of the instruments require a license.

### Dependent variables

#### Job Strain

##### Strain in dementia care scale (SDCS)

The SDCS is a self-reported instrument and addresses the perceived level of job strain among care staff. The SDCS was developed by a multi-country research group to explore nurses’ experience of strain when working in dementia care [10, 31]. SDCS has later been used for care staff working with older adults with and without dementia in residential nursing homes and home care settings [6, 13, 31, 32]. The aim of the SDCS is to identify the level of job strain within different aspects of the work environment. The instrument’s developer refers to Knapp´s definition of strain “as the effects of stress ‘the wear and tear itself’”, but also explains that the causality relationship between stress and strain are inter-related with a complex system of variables associated to stress [32].

The SDCS consists of 27 statements concerning the care staffs’ work situation. For each statement (item), two aspects are investigated; 1) how frequently the situation occurs and 2) how much stress each situation generates. Both aspects are measured on a four-point Likert scale; 1 = never/no stress to 4 = very often/high stress. The 27 statements are allocated into one of the following five factors: *Frustrated empathy*–F1 (7 items), *Difficulty understanding and interpreting*–F2 (7 items), *Balancing competing needs*–F3 (5 items), *Balancing emotional involvement*–F4 (4 items), and *Lack of recognition*–F5 (4 items) [31].

The level of perceived job strain is calculated by multiplying the response of frequency and stress for each statement which creates an output between 1–16. A higher number indicates a higher perception of job strain [6, 13, 31, 32]. The score of the total job strain, and for each factor, is calculated by adding the scores of each included statement and then dividing the sum with the number of statements included in the whole SDCS, or within each factor [31].

Psychometric properties for SDCS have been deemed acceptable. Internal consistency is valid at α 0.91 to α 0.94 for the whole SDCS and between α 0.53 to α 0.90 for the five factors [6, 13, 31, 32]. No cut-off scores exist for the SDCS; however, previous research presents mean scores between 2.7 and 6.85 [6, 8, 13, 32]. In the present study, a small adaption of the instrument was made to cohere with the HCS´s work environment, and the word *resident/client* was changed to *older adult,* which has been done previously [6].

In this study, the total score of SDCS (referred to as the total job strain) and the sum scores of each SDCS factor (F1-F5) is respectively represented as a dependent variable (DV), giving a total of six DV’s. There is one MLR model for each one of the six DV’s.

### Independent variables

Independent variables are divided into individual and organisational factors. Individual factors consist of characteristic data of the participating HCS, and the HCS’s self-related health, while the organisational factors consist of psychosocial factors at work. Every variable from the individual and organisational factors are considered potential independent variables in the forthcoming MLR models.

#### Individual factors

##### Characteristic data

Characteristic data of the participants included information such as gender, age, Swedish as a first language, formal education, education in care/caring, work time, permanent position, and years of work experience within home care services.

##### Satisfaction with work questionnaire (SWQ)

The SWQ is an instrument that assesses the work environment for staff who are working with care for older adults [33]. Four questions from the SWQ subscale “*psychosomatic health aspect*” (PH) were extracted and included in the survey. These four PH questions focus on the staffs’ experiences during the last 90 days regarding; *1. feeling unhappy and depressed*, *2. having sleep problems*, *3. feeling worried and restless*, and *4. feeling physically fatigued after work*. The responses are rated on a five-point Likert-scale with the options 1 = very often to 5 = never [33].

#### Organisational factors

##### General Nordic questionnaire for psychological and social factors at work (QPSNordic)

The QPS_Nordic_ measures the psychosocial factors of the work environment and is developed from organisational theories to investigate the relationship between work, health and productivity [34]. It is a general self-assessed instrument that evaluates an employee’s perception of their psychological and social work-life in combination with the organisational work relationships within a person’s work-life [35].

The QPS_Nordic_ questionnaire consist of 118 work-related items [35, 36]. In the present study, the shorter version of QPS_Nordic_^34+^, consisting of 37 questions, was used. The first 34 questions are assembled into eight subscales: *Job demands* (4 items), *Role expectations* (3 items), *Control at work* (6 items), *Predictability at work* (1 item), *Mastery of work* (1 item), *Social interactions* (4 items), *Leadership* (2 items), *Organisational culture & climate* (8 items), and *Perception of group work* (2 items). Responses to questions 1–34 are made on a five-point Likert-scale, ranging from 1 = very seldom/never to 5 = quite to very often/always. The last three questions (35 to 37) are not included in any subscale and treated as single items and have the response options 1 = do not agree/not at all to 5 = fully agree/very much. The response frequency is presented in percentages for each item or as a reduced three-scale, where responses 1 and 2, as well as 4 and 5, are combined, or as mean scores for each subscale of QPS_Nordic_^34+^ [34–36]. Limited evidence exists for the validity of the QPS_Nordic_^34+^ subscales [37, 38].

In this study, we chose to treat the single item number 37 as an individual factor instead as an organisational factor since the question is not formulated as a stress situation at work but rather stress on a more general basis.

### Data collection

Data were collected with one web-based and one paper questionnaire with the following structure: characteristic information, the SDCS, the four PH questions, and the QPS_Nordic_^34+^.

Included home care agencies received oral and written information about the project. A consent letter and a link to the web-based survey was sent out to all HCS at these agencies. The web-survey was accessible between April to June 2018 and consent was given by starting the survey. Due to a low response rate during this period, a paper questionnaire was utilized during September to November 2018. A researcher from the team visited all units and informed participants about the questionnaire. Staff who had participated in the web-based survey were asked not to partake a second time. The staff deposited the completed paper questionnaire in a secure response box at the unit, which was collected after two weeks.

Data from non-responders were not collected.

### Data analysis

All data were checked by the first and third author and transferred to SPSS version 26.0 [39] where all analyses were performed. If participants had changed an answer by crossing over or erasing their response, it was treated as missing if consensus was not reached between the two researchers.

#### Pre-analysis

##### Missing data

Missing data was investigated for the factor groups (F1-F5) in SDCS, the subscales in QPS_Nordic_^34+^, and the PH responses. All five SDCS factors had 20 to 25% missing data; the subscales in QPS_Nordic_^34+^ had 1.8 to 8.4% missing data. Some data was systematically missing; however, most responses were missing at random. A multiple imputation (MI) method was used to replace missing data. MI substitutes values and constricts ambiguity about missing values [40] and improves the validity, increases precision, and enables robust statistics [41]. The outcome of the MI is a mean value that is based on five imputation cases, which is presented in a pooled data set [40]. Missing data from characteristic data was not imputed.

##### Internal consistency of SDCS & QPSNordic34 +

Internal consistency for the total score and factor/subscale scores for SDCS and QPS_Nordic_^34+^ were measured with Cronbach´s Alpha (α), with a cut-of at α 0.70, but with minimal acceptance at α 0.65 to 0.70 [42]. The QPS_Nordic_^34+^ had inconsistent results where only the total QPS_Nordic_^34+^ and four out of nine subscales had valid outcomes (α 0.68 to 0.89): *Job demand*, *Control at work*, *Leadership* and *Perception of group work*. Three subscales were re-built to reach a valid α by testing the sets of items that corresponded to the subscale of the full version of QPS_Nordic_ [38], these were: *Role expectation* (α 0.69), *Social interactions* (α 0.73), and *Organisational culture and climate (*α 0.84). The two subscales *Prediction at work* and *Mastery of work* only consisted of one or two items initially, hence, they were not possible to re-build. All items that in the end did not belong to a subscale were treated as single items.

##### Responses of SWQ

The responses of the SWQ were inverted so that a higher score indicated poorer health.

### Statistics

Descriptive statistics were used for the participants’ characteristics and for all items in each instrument, using frequencies and percentage for categorical variables, and means and standard deviations for continuous variables.

Inferential statistics such as chi-square and ANOVA test were applied for analysing differences of job strain between the categorical variables, multi-choice responses and interval characteristics. Normal distribution and homoscedasticity were examined to determine if a MLR could be conducted. Multicollinearity was applied to ensure basic assumptions for MLR and the included variables, with the cut-off set to *r* < 0.80, VIF < 5, and Tolerance < 0.40 [43].

#### Multiple linear regression analyses

In total, six MLR models were assessed, where total job strain and each of the five SDCS factors (F1 to F5) were used as a DV in each of the six models. Individual factors in terms of characteristics of participants, the four PH questions, and the QPS_Nordic_^34+^ single item 37, as well as organisational factors in terms of subscales and single items in the QPS_Nordic_^34+^, were all considered possible independent variables in all six MLR models. Initial analysis identified eligible independent variables for each forthcoming MLR model. Each independent variable had to meet the criteria for the unstandardized regression coefficient (*B*) p-value at < 0.05, as well as having a significant correlation (*p* = 0.05) with the DV. An Adjusted R^2^ above 0.20 was considered good based on previous research within the field where subjectively perceived data has been used [44].

## Results

The outcomes are presented below in the following order: a description of the sample, descriptive outcomes of the DV’s regarding mean level of perceived job strain from the total SDCS score and the five SDCS factors (F1-F5), as well as from specific items in SDCS. This is followed by outcomes of the individual and organisational independent variables, and finally, the results of the six MLR analyses are presented.

### Description of the sample

Out of a possible 481 staff members, 226 (47%) from 17 units responded to the survey. A majority of the participants were women (80%), had education in care/caring (86%), and had on average worked 13 years within home care (Table 1).

**Table 1:** Characteristics of participants and mean score of total job strain (SDCS), outcomes of the four psychosomatic health aspects, and the QPS single item 37

Variables	Sample N (%)		SDCS mean (SD)
Total	226		4.44 (1.88)
Gender ^a^			
Female	179 (79.6)		4.55 (1.90)
Male	46 (20.4)		3.99 (1.78)
Age ^c^, y			
Min–max	18–67		
Mean [SD]	48.2 [11.35]		
25 / 50 / 74	39 / 51 / 58		
Swedish as first language ^b^			
Yes	128 (59.3)		4.32 (1.86)
No	88 (40.7)		4.54 (1.93)
Formal Education ^a^			
University	45 (20.1)		4.20 (1.97)
High-School/Secondary School	146 (65.2)		4.40 (1.87)
Elementary school	26 (11.6)		5.09 (2.25)
Others	7 (3.1)		3.95 (1.82)
Education in care/caring ^a^			
Yes	193 (85.8)		4.53 (1.95)
No	32 (14.2)		3.87 (1.36)
Work time ^a^			
Fulltime	147 (66.8)		4.42 (1.85)
Part-time	73 (33.2)		4.41 (1.88)
Permanent Position			
Yes	207 (91.6)		4.46 (1.86)
No	19 (8.4)		4.16 (2.18)
Work experience within home care services ^b^, y			
Min–Max	0–40		
Mean [SD]	12,56 [9.24]		
25 / 50 / 75	5 / 11 / 18		
Psychosomatic Health aspects (*n* = 226)	Never/rather seldom	Sometimes	Rather/very often
1. Feeling unhappy & depressed	22.4	40.8	36.8
2. Having sleep problems ^a^	28.4	31.5	40.1
3. Feeling worried & restless	22.9	30.9	46.2
4. Feeling physically exhausted ^a^	56.3	27.9	15.8
QPS _Nordic_^34+^ (*n* = 226)	Not at all/only a little	To some extent	Rather/very much
Item 37 – Feeling stressed	32.3	28.8	38.9

*Note*: Characteristic data presented in n (%) and y = years. Strain of Dementia Care Scale (SDCS) presented with mean and standard deviation (SD) for the total job strain in the whole sample and each characteristic domain except for variables with years. Psychosomatic Health aspects (PH) presented in % with a reduced response scale 1 & 2 = never/rather seldom, 3 = sometimes, 4 & 5 = rather/very often. QPS _Nordic_^34+^ item presented in % with a reduced response scale 1 = not at all to 5 = very much. Missing value for each variable: ^a^0,4 to 2,6%, ^b^4,4 to 7%, ^c^19%

### Descriptive outcomes of the dependent variables

Our first aim was to explore the perceived level of job strain for HCS.

The mean job strain level for the HCS in this study was 4.43 (SD 1.8). Regarding the sub-factors of SDCS, the factor *Lack of recognition* (F5) was rated with the highest mean of job strain, 5.34 (SD 2.67). F5 also includes the item *I want to do much more for older persons than my employers will allow*, which received the highest mean job strain score, 6.55, in the whole SDCS. The factor *Difficulties understanding and interpreting* (F2) received the lowest mean of job strain 3.08 (SD 1.66) and also included the item that received the lowest mean in the whole SDCS, which was *I have difficulties understanding older persons’ needs* (2.69) (Table 2).

**Table 2:** Total job strain and sub-factors (F1 – F5) from the SDCS, with included statements

SDCS	Sample	Min–Max	Mean (SD)	(α)
**Total job strain**	226	1.05—9.99	4.43 (1.88)	0.94
**F1: F*rustrated empathy***	226	1.00—14.86	4.73 (2.26)	0.85
I see other staff behaving toward an older person in ways that show they do not understand the effects of dementia			5.33	
I see that an older person is suffering			5.95	
Older persons do not receive the care I feel they are entitled to			5.79	
I see the families of older persons suffering			4.25	
I see older persons being mistreated by their families			4.04	
I see other staff treating older persons badly			3.26	
Other staff tries to change what I have done for an older person			4.47	
**F2: D*ifficulties understanding and interpreting***	226	1.00—8.29	3.08 (1.66)	0.85
I have difficulties understanding what older persons are thinking			3.70	
I have difficulties understanding what older persons are trying to communicate			2.91	
I have difficulties understanding older persons’ needs			2.69	
I find it difficult to know what is best for older persons			2.97	
I worry I might upset or hurt older persons because I do not understand their needs			3.08	
I cannot understand why older persons behave the way they do			2.90	
I find it difficult to explain to older persons what is happening in situations which may upset them (e.g., showering, bathing, or toileting)			3.28	
**F3: B*alancing competing needs***	226	1.00—11.60	4.53 (2.28)	0.78
I must balance the needs of the older person against the needs of his or her family			4.57	
I must balance the needs of the older person against the needs of other older persons			4.58	
I must prioritize on the basis of urgency rather than fairness or the needs of older persons			5.17	
Older persons resist the care I want to provide			4.55	
I must balance the safety of older persons against their quality of life			3.77	
**F4: B*alancing emotional involvement***	226	1.00—13.50	4.51 (2.31)	0.73
When an older person dies or must move, I feel as though I have lost a relative or close friend			4.09	
I feel that older persons are highly dependent on me			5.71	
I wish I knew more about older persons so that I could understand them better			4.24	
I cannot stop thinking about older persons when I am away from work			3.80	
**F5: *Lack of recognition***	226	1.2 5- 14.25	5.34 (2.67)	0.70
I feel that my work is not valued by others			4.83	
I want to do much more for older persons than my employers will allow			6.55	
My employers do not appreciate the work I’m doing			4.28	
Families of older persons do not seem to understand how difficult it is to care for their relative			5.70	

*Note***:** Scores are ranging between 1–16. Higher scores indicate a higher level of job strain. Cronbach´s Alpha (α)

### Descriptive outcomes of independent variables

#### Individual factors

Descriptive information of individual factors regarding the participants' characteristics, outcomes of the PH questions, and the QPS_Nordic_^34+^ single item 37 are presented in Table 1.

More than 70% of the HCS perceived that they sometimes or very often felt unhappy and depressed (PH1), had problems sleeping (PH2), and felt worried and restless (PH3). In addition, 40% perceived that they often felt rather/very stressed (item 37) (Table 1). No significant differences were found within the different characteristic groups regarding total mean SDCS score.

#### Organisational factors

Four out of seven subscales in the QPS_Nordic_^34+^ were rated with low mean scores, indicating a negative outcome. These four subscales were *Job demand* (2.55), *Leadership* (2.72), *Organisational culture and climate* (2.78) and *Control at work* (2.87). More than 50% of the HCS perceived they had too much to do (item 2), were not able to influence the amount of work (item 10), the work pace (item 11), or decide when to take a break (item 12) (Table 3). In addition, nearly half of HCS perceived not receiving sufficient support from their managers to develop their skills (item 20) or being encouraged to participate in important decisions (item 21), and 55% of the HCS perceived that the managements’ interest in the staff’s health and well-being (item 34) was absent (Table 3).

**Table 3:** QPS_Nordic_^34+^ subscales, single items w/o subscales, item number, and the item wording

**Subscale**	**Item nr**	**Item wording**	**Cronbach’s Alpha**	**Mean**	**SD**	Reduced scale. responses (%)		
			(α**)**			**1&2**	**3**	**4&5**
**Job Demand**			0.69	2.55	0.75			
	1 **↓**	Is your workload irregular so that the work piles up?		3	1.1	28.8	39.4	31.8
	2 **↓**	Do you have too much to do?		3.47	0.99	13.7	35.8	50.4
	3 **↓**	Are your work tasks too difficult for you?		1.75	0.90	76.9	19	3.9
	4 **↓**	Do you perform work tasks for which you need more training?		1.99	1.08	69.4	23	7.5
**Role Expectations**			0.69	3.48	0.72			
	7 **↑**	Have clear planned goals and objectives been defined for your job?		3.76	1.12	12.4	20.8	66.8
	8 **↑**	Do you know exactly what is expected of you at work?		4.41	0.85	4.4	5.3	90.2
**Control at Work**			0.75	2.87	0.78			
	5 **↑**	Are your skills and knowledge useful in your work?		4.20	1.05	7.5	10.1	82.3
	6 **↑**	Is your work challenging in a positive way?		3.56	1.04	7	34.9	53.5
	10 **↑**	Can you influence the amount of work assigned to you?		2.31	1.26	55.7	27.9	16.4
	11 **↑**	Can you set your own work pace?		2.37	1.21	58.8	26.5	17.7
	12 **↑**	Can you decide yourself when you are going to take a break?		2.12	1.20	65	22.1	12.8
	13 **↑**	Can you influence decisions that are important for your work?		2.64	1.18	44.2	34.5	21.3
**Social Interaction**			0.73	3.41	0.94			
	17 **↑**	If needed, can you get support and help with your work from your co-workers?		3.79	1.04	8.8	29	62.4
	18 **↑**	If needed, can you get support and help with your work from your immediate superior?		3.40	1.24	22.1	26.9	50.9
	19 **↑**	Are your work achievements appreciated by your immediate superior?		3.03	1.24	32.7	34.1	33.2
**Leadership**			0.89	2.72	1.14			
	20 **↑**	Does your immediate superior encourage you to participate in important decisions?		2.77	1.21	43.8	30.5	25.7
	21 **↑**	Does your immediate superior help you develop your skills?		2.68	1.21	46	30.5	23.5
**Organisational culture and climate**			0.84	2.78	0.85			
	23 **↑**	What is the climate like in your work unit…?		3.06	1.17	31	31.4	37.6
		…Encouraging and supportive						
	24 **↑**	…Relaxed and comfortable		3.04	1.18	32.7	28.3	38.9
	28 **↑**	Are workers encouraged to think of ways to do things better at your workplace?		3.25	1.04	21.7	35.4	42.9
	29 **↑**	Is there sufficient communication in your department?		3.10	1.17	30.5	28.3	41.2
	33 **↑**	At your organisation, are you rewarded (money, encouragement) for a job well done?		1.79	1.06	78.3	13.7	8
	34 **↑**	To what extent is the management of your organisation interested in the health and well-being of the personnel?		2.44	1.18	55.3	25.7	19
**Perception of group work**			0.68	3.88	0.74			
	26 **↑**	Do you appreciate belonging to your workgroup or team?		3.91	0.96	9.3	20.4	70.4
	27 **↑**	Is your group or team successful at problem-solving?		3.85	0.96	7.5	23.9	68.6
**Single Items w/o subscale**								
	9 **↓**	Do you receive incompatible requests from two or more people?		2.54	1.24	46.4	35.4	18.1
	14 **↑**	Do you know in advance what kind of tasks to expect a month from now?		2.19	1.49	64.6	9.3	26.1
	15 **↓**	Are there rumours concerning changes at your workplace?		2.85	1.15	34	38.9	27
	16 **↑**	Are you content with your ability to solve problems at work?		3.95	0.97	6.6	21.2	72.1
	22 **↑**	Do you feel that your friends/family can be relied for support when things get tough at work?		3.35	1.40	27.4	21.7	50.9
	25 **↓**	What is the climate like in your work unit?—Rigid and rule-based		2.86	1.15	40.2	29.2	30.5
	30 **↓**	Have you noticed any disturbing conflicts between co-workers?		2.92	1.13	35.4	35.4	29.2
	31 **↓**	Have you noticed any inequalities in how men and women are treated at your workplace?		1.97	1.20	73.5	14.2	12.4
	32 **↓**	Have you noticed any inequalities in how older and younger employees are treated at your workplace?		1.92	1.15	73.5	15.9	10.6
	35 **↑**	I like to be absorbed in my job most of the time		3.65	1.09	12.8	28.8	58.4
	36 **↑**	The major satisfaction in my life comes from my job		2.81	1.25	34.5	32.3	33.2
	37 **↓**	Stress means the situation when a person feels tense. restless. nervous. or anxious. or is unable to sleep at night because his or her mind is troubled all the time Do you feel that kind of stress these days?		3.08	1.25	32.3	28.8	38.9

Note: The scale ranges from 1 = very seldom/never to 5 = very often/always for items 1–34, 1 = do not agree to 5 = fully agree for item 35 and 36, and 1 = not at all to 5 = very much for item 37. Mean and SD for each subscale. Mean, SD for each item, and percentage frequency on the reduced response scale for each item. Cronbach´s Alpha (α) for all subscales. ^↑^ Indicates positively formulated questions, where a higher score relates to a positive impact. _↓_ indicates negatively formulated questions, where higher scores relate to a negative impact

A positive outcome in the psychosocial work environment (QPS_Nordic_^34+^) was found for the subgroups *Perception of group work* (3.88), as well as *Role expectation* (3.47). Within these two subscales, the majority of the HCS (70%) rated that they quite often or very often appreciated belonging to their group at work (item 26) and they considered the group to be good at solving problems (item 27). Furthermore, 67% of the HCS reported having well-defined goals for their work (item 7), and 90% knew what was required of them at work (item 8) (Table 3).

### Individual and organisational factors associated with job strain

The second aim of this study was to explore the associations of individual and organisational factors with job strain. The results from the six MLR models are presented in Table 4, showing that both individual and organisational factors are associated with job strain in all six models.

**Table 4:** The six Multiple Linear Regression Models with SDCS total job strain and the five factors (F1 – F5) as dependent variables (bold) with the organisational (Org.) and individual (Ind.) factors that explain the model

	Org. and Ind. factors	Unstandardized Coefficients						R^2^ /Adjusted R^2^
		*B*	SE	T	p-value	95% CI		
**Job Strain (DV)**								.518 / .507
(Constant)		-0.08	0.33	-0.25	0.803	-0.74	0.57	
QPS Job demand	(Org)	0.70	0.16	4.42	0.001**	0.39	1.01	
QPS Item9 *incompatible requests*	(Org)	0.24	0.08	2.88	0.004**	0.08	0.40	
QPS Item 37 *feeling stressed*	(Ind)	0.20	0.10	1.97	0.051***	-0.00	0.41	
PH 1 *feeling unhappy & depressed*	(Ind)	0.30	0.13	2.40	0.017*	0.06	0.55	
PH 3 *feeling worried & restless*	(Ind)	0.24	0.12	2.02	0.045*	0.01	0.48	
**F1 *Frustrated Empathy (DV)***								.406 / .389
(Constant)		0.08	1.13	0.069	0.945	-2.14	2.30	
QPS Job demand	(Org)	0.50	0.21	2.41	0.016*	0.09	0.92	
QPS Role expectation	(Org)	0.38	0.18	2.09	0.036*	0.02	0.74	
QPS Organisational Culture and Climate	(Org)	-0.48	0.17	-2.88	0.004**	-0.81	-0.15	
QPS item 16 *content with one’s ability to solve problems at work*	(Org)	0.32	0.14	2.36	0.019*	0.05	0.58	
QPS item 32 *age inequalities at work*	(Org)	0.36	0.11	3.14	0.002**	0.13	0.58	
PH 3 *feeling worried & restless*	(Ind)	0.54	0.12	4.40	0.001**	0.30	0.79	
**F2 *Difficulty Understanding and Interpreting (DV)***								.207 / .200
(Constant)		0.58	0.36	1.61	0.107	-0.126	1.29	
QPS Job demand	(Org)	0.70	0.16	4.30	0.001**	0.381	1.02	
PH 3 *feeling worried & restless*	(Ind)	0.27	0.10	2.69	0.008**	0.071	0.46	
***F3 Balancing Competing Needs (DV)***								.418 / .399
(Constant)		0.28	1.12	0.25	0.803	-1.92	2.48	
QPS Job demand	(Org)	0.99	0.21	4.75	0.001**	0.59	1.41	
QPS Role expectation	(Org)	-0.83	0.35	-2.42	0.018*	-1.52	-0.15	
QPS Social interaction	(Org)	0.33	0.16	2.10	0.036*	0.02	0.64	
QPS Item 9 *incompatible requests*	(Org)	0.75	0.23	3.35	0.001**	0.30	1.20	
QPS Item 16 *content with one’s ability to solve problems at work*	(Org)	0.31	0.14	2.19	0.028*	0.03	0.59	
QPS Item 35 *like to be absorbed in my job*	(Org)	-0.30	0.13	-2.30	0.022*	-0.57	-0.04	
PH 1 *feeling unhappy & depressed*	(Ind)	0.53	0.13	4.16	0.001**	0.28	0.77	
**F4 *Balancing Emotional Involvement (DV)***								.435/ .427
(Constant)		-0.40	0.44	-.91	0.363	-1.27	0.47	
QPS Job demand	(Org)	0.88	0.21	4.26	0.001**	0.48	1.29	
QPS Item 37 *feeling stressed*	(Ind)	0.46	0.14	3.40	0.001**	0.19	0.73	
PH 3 *feeling worried & restless*	(Ind)	0.47	0.14	3.46	0.001**	0.20	0.73	
**F5 *Lack of Recognition (DV)***								.471 / .456
(Constant)		0.25	1.06	0.23	0.818	-1.84	2.33	
QPS Job demand	(Org)	0.70	0.24	2.91	0.004**	0.23	1.17	
QPS Organisational Culture and Climate	(Org)	-0.40	0.19	-2.06	0.039*	-0.77	-0.02	
QPS Item 9 *incompatible requests*	(Org)	0.30	0.13	2.35	0.019*	0.05	0.56	
QPS Item 37 *feeling stressed*	(Ind)	0.37	0.16	2.29	0.025*	0.05	0.70	
PH 3 *feeling worried & restless*	(Ind)	0.50	0.16	3.20	0.002**	0.19	0.81	
Education Level	(Ind)	0.57	0.21	2.70	0.007**	0.16	0.99	

*Note*: Significant levels are set to: * < 0.05 and ** < 0.01. *** Included for showing clinically significant level

The MLR model with *Job strain* (total SDCS scoring) as the DV, identified five variables that explained 51% of the variance. The organisational factors were *Job demand (subscale)* and *Incompatible requests* (item 9), while the individual factors were *Feeling stressed* (item 37) and the two PH questions related to the perception of *Feeling unhappy and depressed* (PH1) and *Worried and restless* (PH3).

In the MLR model with *Lack of recognition* (F5) as the DV, six variables explained 46% of the variance. F5 addresses HCSs’ desire to do more for their clients and being recognised for their work by the client’s family, their manager, or by others. A higher level of lack of recognition was associated with the organisational factors perceiving incompatible requests from others (item 9) and the two subscales *Job demand* and *Organisational culture and climate*. *Organisational culture and* c*limate* includes aspects such as if one is in a supportive and comfortable work environment, being encouraged to improve things at the workplace, or being rewarded for a well-done job. Additionally, the individual factors of feeling stressed (item 37), feeling worried and restless (PH3), and the level of education, were also significantly associated with a high lack of recognition (Table 4).

The MLR model with *Frustrated empathy* (F1), *Balancing competing needs* (F3) and *Balancing emotional involvement* (F4) as DVs, identified individual and organisational factors that explained 39, 40 and 43% of the variance (Table 4). Finally, the model with *Difficulty understanding and interpreting (F2*) as the DV, had the lowest explanation rating (20%), but included the two most common variables in all MLR models: the organisational factor *Job demand* and the individual factor *Feeling worried and restless* (PH3).

The organisational factor *Job Demand (*subscale*)* was associated with the DV´s in all six MLR models and the individual factor *Feeling worried and restless* (PH 3) was associated with job strain in five out of six MLR models*.* In three out of six MLR models the individual factor *Feeling stressed* (item 37) and the organisational factor *Perception of receiving incompatible requests* (item 9) were associated with job strain.

## Discussion

The aim of this study was to explore how HCS perceive their level of job strain, and how job strain was associated with, and to what extent job strain can be explained by, individual and organisational factors of the psychosocial work environment and psychosomatic health factors. As follows, we will focus the discussion on three areas. First, we will discuss the complexity of job strain, secondly, the most frequent factors in the MLR models that explained job strain, and lastly, strategies that can be implemented to reduce job strain among HCS.

### Complexity of job strain

The results of this explorative study show that HCSs’ perceived job strain is explained by a combination of both individual and organisational factors within the psychosocial work environment. The variety of factors that explain job strain for HCS in this study ranges from perceiving high job demands, organisational culture and climate, and role expectations, to education level and feeling stressed, unhappy, and worried. Other studies have also pointed out that leadership factors, lower competence level, low control of decision-making, and unmet needs are associated with higher job strain and a negative psychosocial environment [6, 7]. Hence, job strain is a multifaceted and a complex concern. Even so, job strain is more complex than just considering the above stated variables. Therefore, we will continue the discussion to acknowledge factors in the context that would be possible triggers to the outcomes of job strain.

#### Contextual factors influencing the complexity of job strain

The contextual factors that potentially influences the HCS job strain lies within the paradigm of today´s eldercare, where aging-in-place with home care is prioritised to living in nursing homes [1, 4, 8]. This has not only created new demands and challenges for the older adult, but also for the HCS [1]. Since older adults who now are living at home are frailer with multiple health conditions, leading to complex health issues, more advanced care and support is required [1, 5, 45]. Hence, this leads to implications for the HCS in regards of an increase of different tasks imposed on the HCS, increased workload, as well as demands of increased competencies [1, 4, 9, 10, 47]. In addition, the HCS have strict time regulations for their support, deficiencies in collaboration with health professions, a staffing shortage, and are not provided with skill development opportunities [11, 46, 47]. Hence, the combination of all these factors reflects the complexity of job strain, as multitude aspects have to be considered in relation to each other.

Because of the present situation, there is a need for changes to ensure a sustainable work environment for HCS. When updating national guidelines and policies for home care systems and developing organizational changes to improve the HCS´s psychosocial work environment, a span of approaches must be taken into consideration, to include both individual and organisational aspects.

### Frequent factors explaining job strain

Since job strain is complex and influenced by a combination of individual and organisational factors, we will address the variables that most frequently explained job strain in this study. These were the two individual factors: *Frequently feeling worried and restless* and *Feeling stressed* and the organisational factors: *Job demands*, *Role expectations*, *Organisational culture and climate, and Perception of receiving incompatible requests.*

#### Job demand and individual factors

The organisational factor of the QPS_Nordic_^34+^ subcategory *Job Demand* was associated with high job strain in all six MLR models (Table 4) and the two individual factors *feeling worried and restless* and *feeling stressed* were found to be associated with job strain in five out of six MLR models. The subfactor *Job Demand* includes questions about irregularity and amount of work as well as one’s competencies to manage the work tasks.

In this study, 70 to 86% of the HCS´s perceived a high job demand, as well as feeling worried, restless, and stressed. These results align with previous Swedish research involving staff in both home care and residential care, who also emphasized that high job demands and feelings of worrying were associated with higher job strain [6, 13, 16, 27]. When not being able to manage or influence your work and not having time for unexpected situations, the combined situation leads to higher job strain for HCS [48] In addition, it has been concluded that the workload for HCS’s many times are too high, being one reason why HCS´s consider quitting their work [49, 50].

#### Job strain and ill-health

A majority of the participants in this study reported that they felt depressed and had sleeping problems, which were strongly associated with perceiving a higher level of job strain. These results are troublesome, as research has shown that a higher job strain increases the probability of depressive symptoms and major depression [18, 51]. The combination of psychosomatic issues, high job strain and demands, low control of work, and low support, are factors leading to a risk for ill-health [16, 51]. Ill-health for HCS can lead to consequences that not only affect the individual but also the recipients of the service as well as the organisation. A newly published report from the Swedish National Board of Health and Well-fare [11] concludes that HCS in Sweden are on sick leave twice as often compared to other professions in the health and well-fare sector. Sick leave influences the stability of staffing, which could lead to decreased quality of care and safety for the older adults who are receiving the service [11]. In addition, attention should be drawn to how the staffs’ health could be viewed as a resource [29, 52]; where a healthy staff can contribute with positive outcomes for the organisation in terms of ensuring staff continuity and thereby providing a safe, efficient service with higher quality to the older adults. Research targeting how working conditions for HCS could influence the delivered care perceived by older adults is limited. However, a newly published Swedish study within this field found a positive association between low job strain for the HCS and older adults’ perception of overall satisfaction, staff treatment and sense of security [53].

### Organisational support and the managers' role

In addition to the organisational factor *Job demand*, the organisational factor *Organisational culture and climate* was also significantly associated with the two MLR models with *Frustrated empathy* (F1) and *Lack of recognition* (F5) as DVs (Table 4), where a negative perception of support and recognition contributed to a higher job strain. Managers who overlook their staff, are not concerned about the staffs’ health, do not enable communication, or have staff that experience an unencouraging and unsupportive environment, can create additional problems to an already strained situation [7, 28, 54]. The independent work and the limited contact with colleagues and managers can further contribute to a more strained situation [55]. In this study, the HCS perceive a lack of continuous and sufficient communication within the work setting and perceived that the management was not interested in their health and well-being. Hence, increased communication and support from managers, as well as from the organisation overall, is crucial for dealing with negative job strain and to promote positive health and well-being among HCS [28]. In the setting of home care, a more active communication is required by managers compared to other workplaces, such as residential care. In home care, there is a lack of spontaneous meetings, which negatively influence the HCSs’ possibility to address issues that occur throughout the day and also the possibilities to receive support from the manager.

The support and leaderships behaviours of the HCSs’ managers are associated with the level of job strain and a negative psychosocial work environment can result in psychological disruptions and long-term sick-leave [7, 13, 14, 28, 56]. By enabling a supportive leadership, as well as a supportive organisation, there may be possibilities to reduce job strain and develop a positive atmosphere that protects against adverse health effects among staff [28].

### Strategies to reduce job strain among HCS

#### Communication and reflection

Efficient strategies to decrease job strain regarding *Frustrated empathy* and *Lack of support* could be to enable time and resources for group sessions with a focus on self-reflection and communication. Previous studies have shown that HCS perceive limited time for self-reflection, developing necessary communication skills, and meeting colleagues before or after their shift, all of which they considered important [5, 48, 57]. Efficient strategies to improve the HCS situation could be to provide and enable time and resources to develop sessions for self-reflection and communication. A supportive relationship among colleagues in combination with sharing and learning together, could build health-promoting relationships and considered to be strategies to improve the psychosocial work environment [29]. Previous studies indicate that intervention programs that focus on knowledge translation regarding evidence-based and person-centred care can be a support to reduce job strain [8].

#### Reablement programs to address job strain in home care

Another strategy to reduce job strain would be to apply a reablement approach, where teamwork, structured planning, and communication is central [58]. Reablement has been tested and implemented in home care organisations in several countries [59–62]. Research has identified that HCS find reablement beneficial for them as their work becomes more efficient, older adults have a more positive attitude towards them, and their roles shift from delivering home care to providing a more person-centred support, reinforcing their roles within health care [57, 63].

Reablement facilitates a structure to the HCS work as the base entails a provision of person-centred care where older adults are empowered to do needed or desired everyday activities by themselves or in collaboration with the HCS, rather than having HCS doing activities for them [59, 64, 65]. In addition, reablement is also a useful approach to incorporate opportunities to share experiences and reflect upon these experiences with colleagues as a part of the work strategy [57, 66]. Reablement has so far only been implemented on a small scale in Sweden, although research is ongoing [66, 67]. However, to learn more about how reablement can support the improvement of, and contribution to, a sustainable work environment for HCS, more research is needed to explore if and how reablement could contribute to reduce job strain for HCS.

### Limitations and strengths

A limitation was the low response rate in this study, which was 47%. Although only one large county was targeted, the 266 respondents were distributed in 5 municipalities, among 5 home care agencies with a total of 17 home care units. However, the participants characteristics: gender, age, years of experience working within home care, having Swedish as a first language, and education level, reflects the population in other studies that has included home care staff in Sweden [6, 54, 56].

With regards to limitations, there was missing SDCS data in this study. Similar issues have occurred in previous studies [6, 13, 31], although it is unclear if data in those studies were missing at random and to what degree. In this study, 20–25% of the SDCS data were missing. This problem concerned the second aspect of the SDCS response option about stress in the specific situation, where the data was mostly missing at random. This could be a design error in the questionnaire, as the two aspects were responded to on the same row as the question, resulting in the possibility that the participant did not observe the need to respond to two different aspects (Supplement 1). Orrung-Wallin et al. [13] had similar problems but considered the questionnaire to provide valuable information although results could be systematically biased. Even if SDCS provides valuable information, systematic bias due to a design fault in a questionnaire is considered problematic for its validity. To deal with the missing data in this study, a MI method was used instead of single imputation as in previous studies [6, 13, 31]. The strength of using MI is that it is considered a more valid and robust procedure where values are randomly replaced by plausible values [68] and account for uncertainties associated with the imputed value [69]. Although, with such a high number of missing data in one aspect, other methods could be considered and evaluated to better manage the problem.

In regards of instruments that assesses the psychosocial work environment for home care staff, other tools such as the COPSOQ [70] are available However, we chose to use the QPS_Nordic_^+ 34^ because of the familiarity of the instrument and the possibility to compare the results of the present study with previous studies in this area.

## Conclusion

This study indicates that there is an intertwined complexity of individual and organisational factors that affect HCSs’ perception of job strain. This complexity requires implementation of new work strategies that are multidimensional, aiming to reduce the level of job strain and thereby create a positive psychosocial work environment for HCS. Future research should focus on implementing reablement in Sweden and explore if reablement can be a method to reduce job strain for HCS.

In addition, the strained situation for HCS implies a pressing need for policy changes within the home care system. Policymakers should focus on developing more appropriate and sustainable strategies to improve the psychosocial work environment for HCS.

## Supplementary Information

**Additional file 1.**

## Data Availability

The datasets used and analysed during the current study are available from the corresponding author on reasonable request.

## Abbreviations

CG: Control group
DV: Dependent variable
F1: Frustrated empathy
F2: Difficulty understanding and interpreting
F3: Balancing competing needs
F4: Balancing emotional involvement
F5: Lack of recognition
FORTE: The Swedish Research Council for Health, Working Life and Welfare
HCS: Home care staff
IG: Intervention group
MI: Multiple imputation
MLR: Multiple Linear Regression
PH 1: Psychosomatic Health 1—feeling unhappy & depressed
PH 2: Psychosomatic Health 2—having sleep problems
PH 3: Psychosomatic Health 3—feeling worried & restless
PH 4: Psychosomatic Health 4—feeling physically exhausted
QPSNordic: General Nordic Questionnaire for Psychological and Social Factors at Work
SDCS: Strain in Dementia Care Scale
SNBHW: Swedish National Board of Health and Well-fare
SWQ: Satisfaction with Work Questionnaire
