# Correction: Individual and organisational factors in the psychosocial work environment are associated with home care staffs’ job strain: a Swedish cross-sectional study

**DOI:** 10.1186/s12913-022-08998-w

**Published:** 2022-12-29

**Authors:** Susanne Assander, Aileen Bergström, Helen Olt, Susanne Guidetti, Anne-Marie Boström

**Affiliations:** 1 Department of Neurobiology, Caring Sciences and Society, Division of Occupational Therapy, Karolinska Institutet, Stockholm, Sweden; 2 Department of Neurobiology, Care Sciences and Society, Division of Nursing, Karolinska Institutet, Stockholm, Sweden; 3 Theme Women´S Health and Allied Health Professionals, Medical Unit Occupational Therapy and Physiotherapy, Karolinska University Hospital, Stockholm, Sweden; 4 Theme Inflammation and Aging, Karolinska University Hospital, Stockholm, Sweden; 5 Research & Development Unit, Stockholms Sjukhem, Stockholm, Sweden

**Correction: BMC Health Serv Res 22, 1418 (2022)**

**https://doi.org/10.1186/s12913-022-08699-4**

Following publication of the original article [1], the authors identified an error in the author names. The given names and family names of all authors were erroneously transposed.

The incorrect author names are: Assander Susanne, Bergström Aileen, Olt Helen, Guidetti Susanne and Boström Anne-Marie

The correct author names are: Susanne Assander, Aileen Bergström, Helen Olt, Susanne Guidetti and Anne-Marie Boström

The author group has been updated above and the original article [1] has been corrected.
