# Supplementary material for: Individual and organisational factors in the psychosocial work environment are associated with home care staffs’ job strain: a Swedish cross-sectional study
Source: BMC Health Serv Res. 2022 Nov 26;22:1418. doi: 10.1186/s12913-022-08699-4 (PMC9701045; doi:10.1186/s12913-022-08699-4)
Supplement: Supplementary file 1 — Additional file 1. [file 12913_2022_8699_MOESM1_ESM.pdf]

Supplement 1. Layout of the SDCS questionnaire.

This is an example of the design of the SDCS questionnaire showing the *item* regarding situations, thoughts, and feelings on the left. The response regarding the *frequency* of these situation, thoughts, and feelings is in the middle, and the response regarding how much *stress* these situations create is on the far right in the questionnaire.
To the left in the picture below, is the original Swedish version used for this study, and to the right a translated English section to illustrate the layout.
Missing data was mostly connected to the question about the level of stress on the right-hand side of the questionnaire.

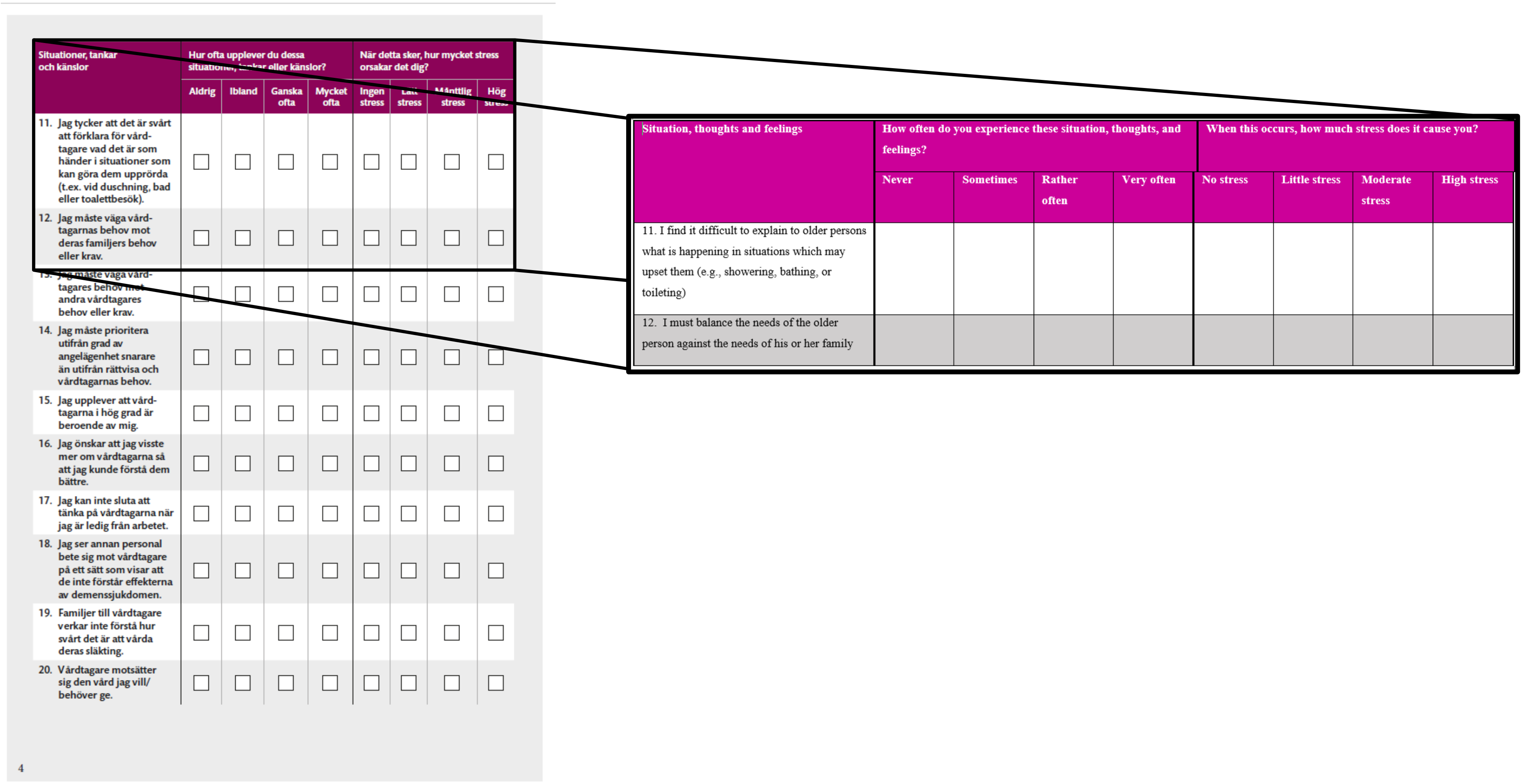

Situationer, tankar och känslor	Hur ofta upplever du dessa situation[illegible] eller känslor?				När detta sker, hur mycket stress orsakar det dig?			
	Aldrig	Ibland	Ganska ofta	Mycket ofta	Ingen stress	[illegible] stress	[illegible] stress	Hög [illegible]
11. Jag tycker att det är svårt att förklara för vård-tagare vad det är som händer i situationer som kan göra dem upprörda (t.ex. vid duschning, bad eller toalettbesök).	☐	☐	☐	☐	☐	☐	☐	☐
12. Jag måste väga vård-tagarnas behov mot deras familjers behov eller krav.	☐	☐	☐	☐	☐	☐	☐	☐
[illegible] vård-tagares behov [illegible] andra vårdtagares behov eller krav.	☐	☐	☐	☐	☐	☐	☐	☐
14. Jag måste prioritera utifrån grad av angelägenhet snarare än utifrån rättvisa och vårdtagarnas behov.	☐	☐	☐	☐	☐	☐	☐	☐
15. Jag upplever att vård-tagarna i hög grad är beroende av mig.	☐	☐	☐	☐	☐	☐	☐	☐
16. Jag önskar att jag visste mer om vårdtagarna så att jag kunde förstå dem bättre.	☐	☐	☐	☐	☐	☐	☐	☐
17. Jag kan inte sluta att tänka på vårdtagarna när jag är ledig från arbetet.	☐	☐	☐	☐	☐	☐	☐	☐
18. Jag ser annan personal bete sig mot vårdtagare på ett sätt som visar att de inte förstår effekterna av demenssjukdomen.	☐	☐	☐	☐	☐	☐	☐	☐
19. Familjer till vårdtagare verkar inte förstå hur svårt det är att vårda deras släkting.	☐	☐	☐	☐	☐	☐	☐	☐
20. Vårdtagare motsätter sig den vård jag vill/behöver ge.	☐	☐	☐	☐	☐	☐	☐	☐

4

Situation, thoughts and feelings	How often do you experience these situation, thoughts, and feelings?				When this occurs, how much stress does it cause you?			
	Never	Sometimes	Rather often	Very often	No stress	Little stress	Moderate stress	High stress
11. I find it difficult to explain to older persons what is happening in situations which may upset them (e.g., showering, bathing, or toileting)								
12. I must balance the needs of the older person against the needs of his or her family								
